# Evaluating Patient Readiness for Community Transition: A Multidisciplinary Audit in Autism Rehabilitation Wards

**DOI:** 10.1192/bjo.2026.11479

**Published:** 2026-06-30

**Authors:** Jhansi Seekolu, Azmathulla Khan, Benny Daniel

**Affiliations:** Cygnet Hospital, Harrow, United Kingdom

## Abstract

**Aims:**

Achieving sustainable community reintegration for autistic adults within specialist inpatient rehabilitation environments necessitates comprehensive, evidence-informed assessment of functional capabilities and adaptive competencies. Autism-specific tertiary rehabilitation units serve a fundamental purpose in cultivating independence, augmenting adaptive repertoires, and ameliorating maladaptive presentations that constitute barriers to successful discharge. This systematic audit investigated the degree to which service users within two Autism Specialist Rehabilitation Wards at Cygnet Harrow Hospital–Springs Centre and Springs Wing–exhibit requisite capabilities for community placement. The principal hypothesis posited that whilst service users would demonstrate substantive advancement across multiple functional trajectories, enduring impediments within domains critical to autonomous functioning would persist, thereby warranting intensified, person-centred therapeutic intervention.

**Methods:**

A comprehensive multidisciplinary audit framework was implemented through collaborative administration by clinical professionals representing occupational therapy, clinical psychology, therapeutic activities coordination, and physical health disciplines. The assessment instrument encompassed 18 clinically validated domains fundamental to rehabilitation progression and discharge preparedness, incorporating activities of daily living, communicative functioning, sensory modulation, adaptive coping mechanisms, affective regulation, interpersonal competence, physical wellbeing, volitional capacity, and overarching integration readiness. Domain-specific ratings were derived through systematic clinical observation, longitudinal therapeutic engagement analysis, and functional performance evaluation, thereby facilitating comprehensive characterisation of individual capabilities and therapeutic needs.

**Results:**

Analytical review identified considerable proficiency across approximately 11 assessed domains, with service users exhibiting sustained progression in self-care competencies, functional communication capacities, emotional self-regulation, therapeutic alliance formation, and diminution of anxiety-driven behavioural manifestations. These therapeutic gains substantiate the efficacy of structured rehabilitative programming and underscore the stabilising influence of coordinated intervention. Conversely, pronounced deficits persisted across six critical areas: capacity for informed autonomous decision-making, behavioural adaptation under stress, social-cognitive competence, preparedness for transition, and proprioceptive awareness alongside motor planning. These domains constitute fundamental prerequisites for discharge viability and emerged as prevalent obstacles throughout the patient cohort.

**Conclusion:**

This audit elucidates that whilst individuals receiving care within autism-specific rehabilitation settings demonstrate meaningful advancement across foundational capabilities, persistent limitations in social cognition, behavioural regulation, and executive decision-making continue to represent significant impediments to placement. Findings illuminate the inherent complexity of preparing autistic adults–particularly those presenting with comorbid intellectual disabilities or challenging behavioural phenotypes–for autonomous living. Outcomes will directly inform collaborative care planning, shape service enhancement initiatives, and facilitate implementation of bespoke therapeutic pathways specifically targeting readiness competencies. Through systematic addressing of identified challenges via empiricallysupported interventions, services can optimise rehabilitative trajectories and substantially enhance prospects for safe, enduring reintegration.

